# DeLLiriuM: A large language model for delirium prediction in the ICU using structured EHR

**DOI:** 10.21203/rs.3.rs-7216692/v1

**Published:** 2025-08-13

**Authors:** Miguel Contreras, Sumit Kapoor, Jiaqing Zhang, Andrea Davidson, Yuanfang Ren, Ziyuan Guan, Tezcan Ozrazgat-Baslanti, Jessica Sena, Subhash Nerella, Azra Bihorac, Parisa Rashidi

**Affiliations:** 1 Department of Biomedical Engineering, University of Florida, Gainesville, FL, USA; 2 Department of Critical Care Medicine, University of Pittsburgh, Pittsburgh, PA, USA; 3 Department of Electrical and Computer Engineering, University of Florida, Gainesville, FL, USA; 4 Division of Nephrology, Department of Medicine, University of Florida, Gainesville, FL, USA; 5 Intelligent Clinical Care Center (IC3), University of Florida, Gainesville, FL, USA

## Abstract

Delirium is an acute syndrome characterized by fluctuating attention, cognitive impairment, and severe disorganization of behavior, which has been shown to affect up to 31% of patients in the intensive care unit (ICU). Early detection can enable timely interventions and improved health outcomes. While artificial intelligence (AI) models have shown great potential for ICU delirium prediction using structured electronic health records (EHR), most studies have either not leveraged state-of-the-art AI models, been limited to single-center cohorts, or relied on small datasets for development and validation. In this study, we introduce DeLLiriuM, a novel LLM-based delirium prediction model that utilizes EHR data from the first 24 hours of ICU admission to estimate a patient’s risk of developing delirium for the remainder of their ICU stay. We developed and validated DeLLiriuM using ICU admissions from 104,303 patients across 195 hospitals in three large databases: the eICU Collaborative Research Database, the Medical Information Mart for Intensive Care (MIMIC)-IV, and the University of Florida’s Integrated Data Repository. Our DeLLiriuM model achieved superior performance compared to all baseline models on the external validation set, measured by the area under the receiver operating characteristic curve (AUROC) metric. DeLLiriuM attained 82.5 (95% confidence interval 81.8–83.1) across 77,543 patients spanning 194 hospitals. Our approach of transforming structured EHR data into an unstructured text format, the primary data modality for LLMs, enables our DeLLiriuM model to capture clinical contextual information, resulting in improved predictive performance. To the best of our knowledge, DeLLiriuM is the first LLM-based delirium prediction tool for the ICU that utilizes structured EHR data with LLMs rather than clinical notes with LLMs or traditional structured feature representations used in AI models.

## 1 Introduction

Delirium is an acute neurocognitive disorder characterized by a fluctuating course, attention deficits, and severe disorganization of behavior^1^. It affects up to 31% of intensive care unit (ICU) patients^2^ and is associated with prolonged ICU and hospital stays, as well as increased ICU and in-hospital mortality rates^3^. Current delirium diagnoses rely on manual assessments such as the Confusion Assessment Method for the ICU (CAM-ICU) and the Intensive Care Delirium Screening Checklist (ICDSC)^4^. While these methods have shown high diagnostic accuracy in the critical care setting^5^, they can only identify delirium after onset. Early detection can facilitate timely interventions and improved patient outcomes.

Multiple studies have developed and validated tools for the early detection of delirium. The PRE-DELIRIC and E-PRE-DELIRIC models, both based on multivariate logistic regression, were designed and externally validated for delirium prediction using risk factors available in the first 24 hours of a patient’s ICU admission^6,7^. Other studies have leveraged predictive features derived from Electronic Health Records (EHR) to forecast delirium at any point during ICU stay using machine learning (ML) classification models^8^. Additionally, dynamic delirium prediction models have also been developed to provide continuous risk assessments 12–24 hours in advance by integrating temporal EHR data (e.g., as vital signs, laboratory test results, assessment scores, and medications) from the preceding 12–24 hours along with static admission data (e.g., age, gender, and comorbidities)^9,10^. Despite these advancements, existing models face several limitations. The PRE-DELIRIC and E-PRE-DELIRIC models were developed using relatively small cohorts (around 3,000 patients in each)^6,7^, limiting their generalization to larger populations. In contrast, studies employing larger cohorts often validate results within a single center^8,10^ or rely on ML and deep learning models (e.g., as Gated Recurrent Unit^11^, Categorical Boosting^12^, and Recurrent Neural Networks^13^) that struggle to capture the long-term dependencies inherent in EHR data^8–10^. This limitation can be mitigated by leveraging state-of-the-art (SOTA) artificial intelligence (AI) models, which have demonstrated superior ability in capturing long-range dependencies^14^.

Large language models (LLMs) have garnered significant interest in the healthcare field^15^. These models, which contain hundreds of millions to billions of parameters, have demonstrated remarkable performance in tasks requiring human language interpretation. To enhance their applicability in medical and clinical tasks, domain-specific LLMs have been developed^16–18^. Notably, their potential for clinical outcome prediction has been explored across various tasks, including in-hospital mortality^19^, heart failure^20^, and ICU length of stay^21^. However, most existing studies rely predominantly on clinical notes written by healthcare professionals. Limited research has investigated the integration of structured EHR data (i.e., table data) in text form, despite evidence suggesting that incorporating structured data into LLMs can enhance predictive performance compared to deep learning models trained solely on structured feature representations^20,22–24^. This gap highlights the potential of LLMs for delirium prediction using structured EHR data. Transforming structured EHR data into an unstructured text format, as opposed to directly providing it in its original structured form, aligns the input with the native processing capabilities of large language models (LLMs). This approach allows the model to leverage the inherent flexibility of natural language, enabling it to capture complex relationships and contextual dependencies that might be overlooked in structured formats. Furthermore, LLMs are inherently designed to process and understand unstructured text, which can enhance their ability to generalize.

In this study, we introduce DeLLiriuM, a novel LLM-based delirium prediction tool that leverages structured EHR data in text form. We develop and validate DeLLiriuM using ICU admissions from 104,303 patients across 195 hospitals within three large databases: the eICU Collaborative Research Database^25^ (hereafter referred to as eICU), the Medical Information Mart for Intensive Care (MIMIC)-IV^26^ (hereafter referred to as MIMIC), and the University of Florida’s Integrated Data Repository (hereafter referred to as UFH). To the best of our knowledge, DeLLiriuM is the first LLM-based delirium prediction tool that relies on structured EHR data.

The study’s main contributions are summarized as follows:

1. We introduce **DeLLiriuM**, a novel LLM-based delirium prediction model that leverages structured EHR data from the first 24 hours of ICU admission to estimate the probability of delirium occurrence throughout the remainder of the ICU stay.
2. We developed an end-to-end pipeline for converting structured EHR into a text report format compatible with LLM models.
3. We propose a novel interpretability framework for text classification outputs, ensuring compatibility with LLM models.

## 2 Methods

### 2.1 Data and Study Design

In this study, three databases were used: UFH, MIMIC, and eICU (cohort diagrams in 1). All data were collected retrospectively. The UFH dataset was retrieved from the University of Florida (UFH) Integrated Data Repository and included adult patients admitted to the ICUs at the University of Florida, between 2014 and 2019. The MIMIC dataset, which is publicly available, was collected at the Beth Israel Deaconess Medical Center from 2008 to 2019^26^. The eICU dataset contains data from ICU patients in 208 hospitals across the Midwest, Northeast, South, and West regions of the United States from 2014 to 2015^25^. In all three datasets, patients under 18 years of age were excluded. In addition, ICU admissions were excluded if they were not the first ICU admission for that patient and/or if the length of stay was less than 24 hours. These criteria were intended to mitigate potential biases toward higher delirium risk during subsequent ICU admissions and to ensure sufficient data were available for prediction. To prevent bias toward predicting higher delirium risk among patients with greater acuity upon admission, we excluded those who died within 48 hours of admission or presented with delirium or coma in the first 24 hours of their ICU stay. Finally, any ICU admission without available EHR data for the first 24 hours was omitted. The UFH dataset was used for training, tuning, and internal validation sets. The MIMIC and eICU datasets served as external validation sets to assess the model’s generalizability across diverse hospital settings. The cohort selection process is described in 1.

### 2.2 Outcomes and Features

The primary outcome predicted by our DeLLiriuM model is the risk of developing delirium at any point after 24 hours of ICU admission. The occurrence of delirium is defined as a positive CAM-ICU score along with a Richmond Agitation Sedation Scale (RASS) score of −3 or higher^4^ at any 12-hour interval after 24 hours of ICU admission (2).

To predict delirium onset, both temporal and static patient information were used as predictive features. The temporal data, extracted from the first 24 hours of ICU admission, comprised four categories: vital signs, laboratory test measurements, medications, and assessment scores (2). Static data included demographic and comorbidity information and was obtained from patient admission records (2). A total of 81 predictive features, common in all study cohorts, were used for delirium prediction. A comprehensive list of the variables used for prediction can be found in Supplementary Table S1.

### 2.3 Model Development and Performance

The DeLLiriuM model was trained on 80% of the UFH dataset, with the remaining 20% split equally for hyperparameter tuning (10%) and internal validation (10%). The data partitioning was performed randomly while ensuring that no patient’s data was included in more than one partition. Data processing for DeLLiriuM involved generating a text-based report from both the static and temporal variables. The DeLLiriuM model used the GatorTronS^27^, a clinical LLM with 345 million parameters and a 512-token context window, as the backbone. GatorTronS was selected based on its superior performance across all cohorts when compared to other large language models (including GatorTron-8b^18^, Meditron-7b^16^, and LLaMa 3–8b^28^). To accommodate the 512-token limit, each temporal variable was summarized by its minimum and maximum values. For medications, the total dose administered in the first 24 hours was used. Then the static data was added at the beginning of the report (2). For the training process, domain-specific pre-training was first conducted using Masked Language Modelling (MLM)^29^ on the summarized EHR text reports from the training set. During this stage, we used Optuna library^30^ to optimize the hyperparameters, which include batch size, learning rate, and number of layers to be frozen/tuned during training. A total of 20 training trials, each running for 100 epochs, were conducted, and the best model (i.e., epoch with the best AUROC) was loaded at the end. The trial with the lowest loss on the tuning set resulted in the domain-specific pre-trained model. Next, the model was fine-tuned for the delirium classification task by freezing all layers except the last 5 (obtained through hyperparameter tuning) and adding a classification head (2). Similar to the pre-training phase, we performed hyperparameter tuning for the same set of hyperparameters for fine-tuning. Twenty trials were run for up to 30 epochs each, and the checkpoint yielding the highest AUROC on the tuning set resulted in the final DeLLiriuM risk inference model. The full hyperparameter search space and final values for both pre-training and fine-tuning are listed in Supplementary Table S2. Two categories of baseline models were used for comparison: structured EHR and text EHR. For the purpose of this study, structured EHR refers to the use of EHR table data in structured feature representations. In contrast, text EHR refers to the same table data converted to a text report as previously mentioned. The structured EHR baselines consisted of one machine learning and four deep learning models: Categorical Boosting (Catboost)^12^, Neural Network (NN), ConCare^31^, Transformer^32^, and Mamba^33^. The Catboost and NN models employed five statistical features (i.e., mean, standard deviation, minimum, maximum, and missingness indicator) to convert the temporal data into a static representation and concatenate with the static data. The ConCare baseline was adapted from the original model^31^ to use the available features, with the use of feedforward and mean imputation for time-series data and mean imputation for static data. The Transformer and Mamba baselines were adapted from previous clinical prediction architectures^14,34,35^, converting time-series measurements into token embeddings, which were then fused with static data. Each deep learning model’s hyperparameters were tuned using the Optuna library^30^, while Catboost hyperparameters (learning rate, number of trees, depth of trees, and L2 regularization) were tuned through grid search. The text EHR baselines consisted of seven LLM models: ModernBERT^36^, ClinicalBERT^17^, GatorTron-8b^18^, GatorTronS^27^, LLaMa 3–8b, LLaMa 3.1–8b, and LLaMa 3.2–1b^28^. Each LLM was fine-tuned only on classification following the same strategy used by DeLLiriuM. All models were trained using an early stopping approach to avoid overfitting.

### 2.4 Model Interpretability

To interpret the DeLLiriuM model predictions, we employed SHapley Additive exPlanations (SHAP) analysis^37^. Since the model’s input consists of text, summarizing the importance of numerical feature values poses a challenge. To address this, we introduced a novel approach for applying SHAP analysis to text-based classification. First, the SHAP analysis was conducted on the full EHR text reports. Then, each report was segmented by feature using the ‘[SEP]’ token, and a corresponding label was assigned to each feature. We computed the sum of the absolute SHAP values for each section and then averaged these values across all samples. The resulting mean SHAP values were used to generate a bar plot, as illustrated in 3. Additionally, we visualized SHAP text plots for four randomly selected cases, representing both positive and negative delirium examples with low and high scores predicted by DeLLiriuM.

### 2.5 Statistical Analysis

To evaluate the statistical significance of the performance differences between the baseline models and the DeLLiriuM model, we compared their AUROC values using the Wilcoxon rank-sum test^38^. A 200-iteration bootstrap was conducted to estimate the 95% confidence interval (CI), with the median AUROC across all iterations serving as the overall performance metric.

## 3 Results

### 3.1 Patient Characteristics

Patient characteristics for this study are presented in Table 1 across all cohorts in terms of ICU admissions. Since only the first ICU admission per patient was considered, the number of ICU admissions corresponds to the number of unique patients. Delirium incidence, as defined by this study’s diagnostic criteria, was 3.6% in UFH (982 patients), 6.8% in MIMIC (1,502 patients), and 2.1% in eICU (1,189 patients), resulting in an overall incidence of 3.5% (3,673 patients).

Across all cohorts, patients with delirium tended to have a higher median age, lower body mass index (BMI), longer ICU stays, and increased rates of coma and mortality compared to those without delirium. In terms of comorbidities in delirious patients, the UFH cohort showed higher chronic heart failure (CHF), renal disease, and liver disease rates. The MIMIC cohort showed higher chronic obstructive pulmonary disease (COPD), cerebrovascular accident (CVA), and liver disease rates. The eICU cohort showed higher CVA and human immunodeficiency virus (HIV) rates.

### 3.2 Model Performance

**Figure 4.**
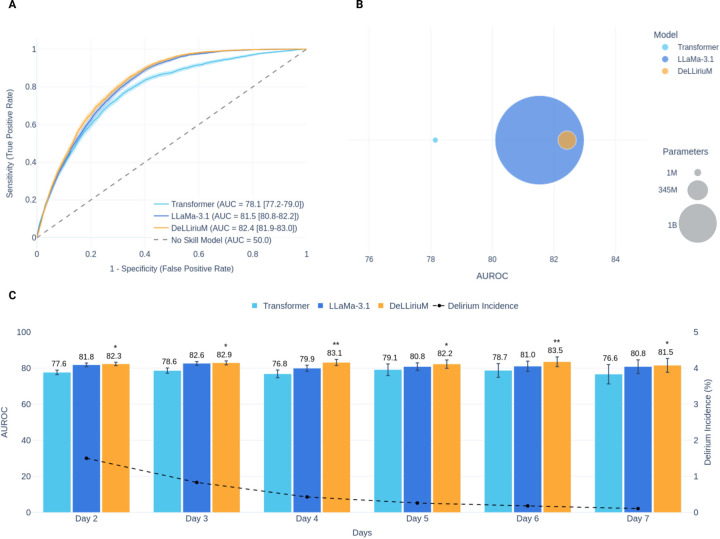
DeLLiriuM performance comparison to best baselines: (A) ROC curves, (B) AUROC relative to model size, (C) AUROC stratified by day in the ICU, along with delirium incidence per day. Note: Confidence intervals and error bars are calculated across 200-iteration bootstrap. Annotations: * p-value <0.05 compared to one baseline. ** p-value <0.05 compared to both baselines.

The performance of DeLLiriuM in terms of AUROC compared to all baseline models is shown in Table 2. The best structured EHR baseline was the Transformer model, with AUROC values of 84.5 (95% CI 79.9–87.6) and 78.1 (95% CI 77.4–78.9) in internal and external validation sets. The best text EHR baseline was Llama 3.1–8b model with AUROC values of 85.5 (95% CI 82.0–88.3) and 81.5 (95% CI 81.0–82.1), respectively. The DeLLiriuM model showed higher performance than both baselines in the external validation set, with an AUROC value of 82.5 (95% CI 81.8–83.1). Although the DeLLiriuM model showed lower performance in internal validation compared to other baselines (84.8, 95% CI 81.8–87.8), this difference was not statistically significant. Figure 4 presents a performance comparison between the DeLLiriuM model and the best baseline models. As shown, DeLLiriuM demonstrated statistically significant higher performance than both baselines on most days. Additionally, when comparing performance relative to model size (i.e., number of parameters), DeLLiriuM maintained strong predictive capability while preserving a lightweight architecture.

### 3.3 Feature Importance

**Figure 5.**
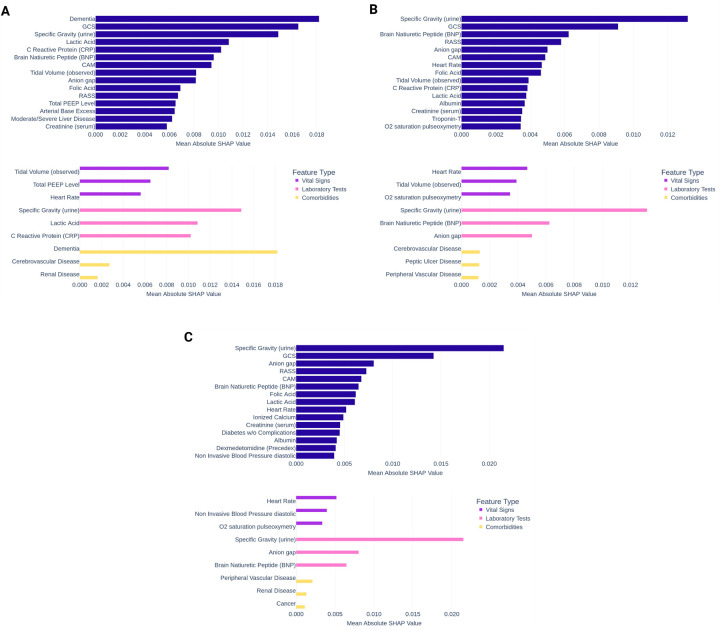
SHAP analysis of DeLLiriuM on (A) UFH, (B) MIMIC, and (C) eICU cohorts.

**Figure 6.**
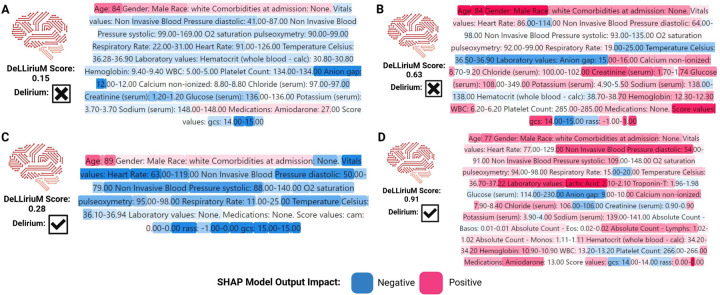
DeLLiriuM predictions for negative examples with (A) low and (B) high predicted scores, and positive examples with (C) low and (D) high predicted scores.

Figure 5 presents the top 15 features for delirium prediction in each dataset based on absolute mean SHAP values, along with the top features in three subcategories: vital signs, laboratory results, and comorbidities. Among the common features in the three datasets, laboratory tests such as specific gravity of urine, brain natriuretic peptide (BNP), and anion gap consistently ranked among the most important. Ventilator settings, including total positive end-expiratory pressure (PEEP) level and observed tidal volume, also ranked highly. Additionally, vital signs such as heart rate, blood pressure, and oxygen saturation exhibited high SHAP values. Figure 6 illustrates SHAP text plots for four randomly selected samples from the eICU cohort, where blue gradients indicate factors negatively associated with delirium, while red gradients represent positive associations. The DeLLiriuM model identified elevated creatinine and lactic acid levels, advanced age, abnormalities in vital signs, and more negative RASS scores (i.e., higher levels of sedation) as strong predictors of delirium. Conversely, high Glasgow coma scale (GCS) scores, normal vital signs and laboratory values, and the absence of certain medications were associated with a lower likelihood of delirium.

## 4 Discussion

### 4.1 Main Findings and Interpretation

The results presented in this study show the development and validation of DeLLiriuM as a tool for predicting delirium in ICU patients. To the best of our knowledge, this is the first study to utilize structured EHR data in text form, alongside large language models, for ICU delirium prediction. Previous studies predicting delirium using EHR data from the first 24 hours of ICU admission have reported varied performance. The *PRE-DELIRIC* model^6^, developed on 1,613 patients from one hospital, achieved an AUROC of 84 (95% CI 82–87) in an external validation on 894 patients across four hospitals in the Netherlands. The *E-PRE-DELIRIC*model^7^, developed on 1,962 patients from 13 ICUs across seven countries, achieved an AUROC of 76 (95% CI 73–78). Additionally, a study that developed a delirium prediction model using eICU data and validated it externally with MIMIC data reported AUROCs of (95% CI 77–80) and 81 (95% CI not reported) on both datasets, respectively. Furthermore, a systematic review of ICU delirium prediction models revealed that AUROC varied greatly (from 62 to 94) across 23 different prediction models^39^. These studies have employed a varying number of features (anywhere from tens to hundreds of features) and a wide range of models (from logistic regression to deep learning models). While drawing direct comparisons between the performance of DeLLiriuM and these models is challenging due to the variation in development and validation settings, the results reported in this paper are comparable and even outperform what is seen in the literature. Notably, this study validates the model on over 100,000 patients, the largest cohort among these studies, achieving strong performance in an external validation set spanning 194 hospitals, despite being trained on data from a single institution. Furthermore, DeLLiriuM leverages state-of-the-art (SOTA) AI techniques, including LLMs and domain-specific pretraining with masked language models. A few studies have employed LLMs with structured EHR data in text form for these tasks and have all seen improved performance compared to structured features deep learning approaches^20,23^. These results are consistent with the results obtained in this work, where DeLLiriuM outperformed structured features (i.e., structured EHR) deep learning approaches. This higher performance could be attributed to the large number of parameters that LLMs possess as well as the large corpus of text that they have been exposed to in their pre-training phase^20,23^.

To further interpret model predictions, SHAP analysis was conducted on the three validation cohorts, revealing that feature importance aligns with established delirium risk factors. Acute hypoxemic respiratory failure (low blood oxygen levels) and acute hypercapnic respiratory failure (high blood carbon dioxide levels), particularly in mechanically ventilated patients, are significant risk factors associated with delirium development in ICU patients^3,40^. As a result, the physiologic parameters depicting signs of respiratory failure—low tidal volumes, higher PEEP levels, lower oxygen pulse oximetry levels, and high end-tidal *CO*_2_ (Et*CO*_2_)—were significant features to predict delirium across the three cohorts. Similarly, sepsis and septic shock due to pneumonia, urinary tract infections, and bloodstream infections are well-known etiologic factors in ICU delirium development. Septic patients typically present with tachycardia (high heart rates), have elevated lactic acid and C-reactive protein (CRP) levels, and sometimes elevated anion gaps, thereby explaining the importance of these features in the three cohorts^3,40^. Patients with systemic conditions like dementia, diabetes, liver disease, and renal disease are highly predisposed to develop delirium^3,40^. Interestingly, abnormal urine specific gravity emerged as a consistently important laboratory feature for delirium prediction across all three cohorts despite not being widely recognized as a conventional risk factor. This association may be due to underlying metabolic abnormalities (e.g., imbalances in sodium, calcium, magnesium, glucose, or dehydration)^41^ as well as urinary tract infection^42^, both of which can contribute to confusion, altered mental status, and delirium development. Notably,^43^ found that urine specific gravity greater than 1.010 predicted early neurologic deterioration in patients with acute ischemic stroke. Another study by^44^ concluded that urine specific gravity above 1.030 was a statistically significant factor for delirium development in post-operative general surgery patients. To illustrate DeLLiriuM’s decision-making process, four representative cases from the eICU cohort highlight its ability to detect known delirium risk factors (6). The DeLLiriuM model was able to correctly identify positive predictors of delirium, such as advanced age, elevated laboratory values such as creatinine and lactic acid, and abnormalities in vital signs, such as low diastolic and systolic blood pressure. On the other hand, it identified negative predictors of delirium development, including normal laboratory results, normal physiologic values (GCS, oxygen saturation), and vital signs (heart rate, blood pressure). Two of the examples show cases where DeLLiriuM predicted a high score (0.63) for a patient who did not develop delirium and a low score (0.28) for a patient who developed delirium. In the first case, the patient had clear risk factors for delirium, such as advanced age, elevated creatinine levels, and low hemoglobin, which can explain the high predicted score. In the second case, the patient had no reported laboratory values and typical values for some vital signs, which can also explain the low predicted score. These findings are all consistent with the literature^40^. While some inconsistencies were observed in the SHAP analysis—such as a GCS score of 14 being a positive predictor in one case but a negative predictor in another—these variations likely reflect complex feature interactions that influence delirium risk. Understanding these interactions will be crucial for further refining DeLLiriuM and improving its interpretability in clinical settings.

### 4.2 Limitations and Future Work

While DeLLiriuM demonstrates strong performance, certain aspects warrant further exploration. First, the short context length of GatorTronS (512 tokens) limits the amount of information that can be incorporated into the EHR text report. Out of the tested models, LLaMa 3, 3.1, and 3.2 had longer context lengths (2,048 tokens). However, the overall performance of these models did not exceed that of DeLLiriuM. This could be explained by GatorTronS being a clinical LLM trained on a large corpus of clinical text, whereas the LLaMa models are general-purpose LLMs. Future experiments will include the use of other domain-adapted LLMs with longer context lengths as well as the use of approaches such as FlashAttention^45^. The summarization method used in this study could also be refined. Reducing each temporal feature to only its minimum and maximum values may exclude important temporal dynamics. Future work will investigate alternative summarization approaches, including multimodal models that integrate text and structured features^46^, as well as the use of LLMs with larger architectures, such as LLaMa3.3–70B and GPT-4o^47^, to generate more comprehensive summaries. Finally, the development and validation of DeLLiriuM were conducted retrospectively. Therefore, future work will focus on validating the model in prospective cohorts to assess its performance in real-world clinical settings. Furthermore, the DeLLiriuM model will be extended to support continuous risk prediction, enabling real-time monitoring of patients’ mental status and enhancing its clinical applicability.

## 5 Conclusion

In summary, we have retrospectively developed and validated DeLLiriuM, an LLM-based model for predicting delirium in ICU patients after the first 24 hours of admission. DeLLiriuM outperforms deep learning models that utilize comprehensive sequential EHR data, despite relying only on a summarized representation of the sequential data. This further underscores the proficiency of LLMs in capturing the nuances and subtleties present in the clinical data. DeLLiriuM enables the screening of patients for delirium risk and provides clinicians with helpful information to make timely interventions. Furthermore, we proposed an automatic text report generation method for structured EHR data, a novel approach for text classification interpretability, and training procedures for clinical outcome predictions using LLMs that can be applied to other prediction tasks. The DeLLiriuM model will be validated prospectively to measure its performance in real-world settings and extended to continuous risk prediction, providing real-time monitoring of patients’ mental status.

## Supplementary Material

Supplementary Files

This is a list of supplementary files associated with this preprint. Click to download.

- deliriumllmscientificreportssupplementary.pdf

## Figures and Tables

**Figure 1.**
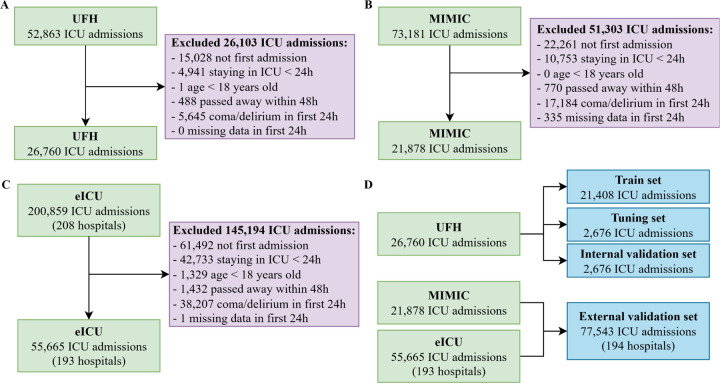
Cohort flow diagram illustrating the inclusion and exclusion processes for each dataset. Panels show (A) eICU, (B) MIMIC, and (C) UFH cohorts, and (D) the final consolidated dataset used in the analysis.

**Figure 2.**
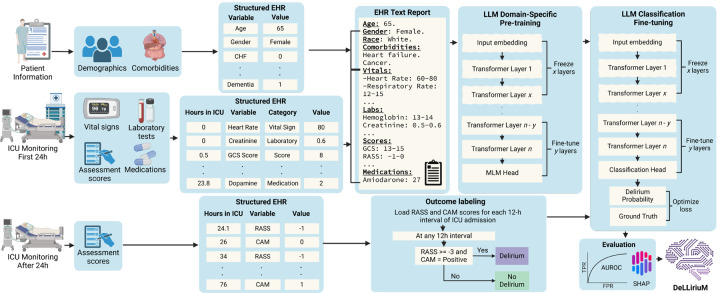
DeLLiriuM model development overview. Data from three different Intensive Care Unit (ICU) datasets were used for training and validating the DeLLiriuM model (195 hospitals, 104,303 patients). Each dataset consisted of ICU monitoring data (i.e., vital signs, medications, laboratory test results, and assessment scores) and patient admission information (i.e., patient demographics and comorbidities). Two assessment scores were extracted for assessment of delirium after the first 24 hours of ICU admission: RASS and CAM. Delirium was defined as any 12-hour interval in which the lowest RASS score was greater or equal to −3 along with at least one positive CAM score. Temporal data from the first 24 hours of ICU monitoring was summarized by taking the minimum and maximum values (i.e., range) of each variable and converted to text along with static patient admission data. GatorTronS^27^ was used as the backbone for the model, which was first pre-trained on the generated EHR text reports with a masked language modeling (MLM) objective and then fine-tuned with a classification objective for delirium prediction. Finally, the model was evaluated using Area Under the Receiver Operating Characteristic (AUROC) and SHapley Additive exPlanations (SHAP) analysis for interpretability, yielding the final DeLLiriuM model.

**Figure 3.**
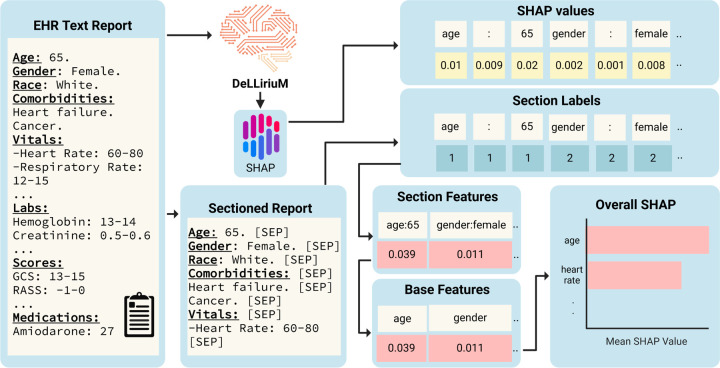
SHAP analysis approach for DeLLiriuM model. The model predictions were interpreted using SHAP analysis. To obtain the overall SHAP value of each feature, text reports were sectioned using the ‘[SEP]’ token and a label was generated for each section. The sum of the absolute SHAP values for each section was computed, and the mean SHAP value across all samples for each feature was taken.

**Table 1.** Patient Characteristics

Cohort	UFH		MIMIC		eICU	
Item	Non-Delirium (n = 25,778)	Delirium (n = 982, 3.6%)	Non-Delirium (n = 20,376)	Delirium (n = 1,502, 6.8%)	Non-Delirium (n = 54,476)	Delirium (n = 1,189, 2.1%)
Hospitals, n	1	1	1	1	193	45
Age, years, m (IQR)	62.0 (49.0–72.0)	67.0 (57.0–76.0)*	65.0 (52.0–77.0)	70.0 (59.0–81.0)*	66.0 (54.0–77.0)	70.0 (58.0–81.0)*
Female, n (%)	11,845 (46.0%)	391 (39.8%)*	9,613 (47.2%)	693 (46.1%)	25,404 (46.6%)	558 (46.9%)
BMI, kg/m2, m (IQR)	27.4 (23.4–32.4)	26.6 (23.2–31.4)*	27.5 (23.9–32.2)	27.1 (23.3–31.6)*	27.7 (23.7–33.0)	26.8 (22.8–31.6)*
ICU LOS, days, m (IQR)	3.0 (1.9–5.1)	9.1 (5.2–15.0)*	2.0 (1.4–3.1)	5.9 (3.5–9.8)*	2.1 (1.5–3.5)	4.9 (2.9–8.6)*
CCI, m (IQR)	2.0 (0.0–3.0)	2.0 (1.0–4.0)*	3.0 (1.0–5.0)	3.0 (2.0–5.0)*	1.0 (1.0–2.0)	1.0 (1.0–2.0)
Race, n (%)						
Black	4,246 (16.5%)	129 (13.1%)*	2,000 (9.8%)	138 (9.2%)	6,398 (11.7%)	187 (15.7%)*
White	19,806 (76.8%)	794 (80.9%)*	14,321 (70.3%)	1,017 (67.7%)	42,240 (77.5%)	886 (74.5%)*
Other	1,726 (6.7%)	59 (6.0%)	4,055 (19.9%)	347 (23.1%)*	5,838 (10.7%)	116 (9.8%)
Comorbidities, n (%)						
CHF	6,182 (24.0%)	304 (31.0%)*	1,616 (7.9%)	139 (9.3%)	1,931 (3.5%)	42 (3.5%)
COPD	7,157 (27.8%)	280 (28.5%)	1,476 (7.2%)	139 (9.3%)*	1,952 (3.6%)	36 (3.0%)
CVA	3,625 (14.1%)	155 (15.8%)	613 (3.0%)	83 (5.5%)*	2,116 (3.9%)	61 (5.1%)*
Malignancy	1,607 (6.2%)	55 (5.6%)	407 (2.0%)	23 (1.5%)	168 (0.3%)	2 (0.2%)
HIV	138 (0.5%)	5 (0.5%)	39 (0.2%)	5 (0.3%)	7 (0.0%)	2 (0.2%)*
Renal disease	4,698 (18.2%)	244 (24.8%)*	1,116 (5.5%)	93 (6.2%)	1,295 (2.4%)	23 (1.9%)
Liver disease	2,034 (7.9%)	131 (13.3%)*	625 (3.1%)	65 (4.3%)*	218 (0.4%)	6 (0.5%)
Outcomes, n (%)						
Coma	1,182 (4.6%)	253 (25.8%)*	873 (4.3%)	514 (34.2%)*	3,070 (5.6%)	238 (20.0%)*
Mortality	581 (2.3%)	125 (12.7%)*	837 (4.1%)	286 (19.0%)*	1,235 (2.3%)	75 (6.3%)*

Abbreviations: BMI: Body Mass Index; CCI: Charlson Comorbidity Index; CHF: Congestive Heart Failure; COPD: Chronic Obstructive Pulmonary Disease; CVA: Cerebrovascular Accident; HIV: Human Immunodeficiency Virus; IQR: interquartile range; LOS: length of stay; m: median.

* P-value <0.05. P-values for continuous variables are based on pairwise Wilcoxon rank sum test. P-values for categorical variables are based on pairwise Pearson’s chi-squared test for proportions.

**Table 2.** DeLLiriuM performance compared to baseline models

Modality	Model	Internal Validation Cohort (UFH)	External Validation Cohort (MIMIC+eICU)
EHR-S	Catboost	84.1 (80.9–87.4)	77.2 (76.5–78.0)
	NN	84.5 (81.0–88.0)	71.7 (70.8–72.7)
	ConCare	72.8 (67.8–77.3)	60.6 (59.6–61.6)
	Transformer †	84.5 (79.9–87.6)	78.1 (77.4–78.9)
	Mamba	83.1 (79.7–86.0)	72.3 (71.5–73.3)
EHR-T	ModernBERT	82.2 (78.4–85.6)	71.9 (71.1–72.7)
	ClinicalBERT	81.9 (78.3–85.2)	78.4 (77.4–79.1)
	GatorTronS	83.3 (79.4–86.2)	79.3 (78.7–80.1)
	GatorTron-8b	84.6 (81.4–87.4)	78.2 (77.4–78.9)
	Llama 3–8b	84.0 (80.7–87.4)	78.4 (77.6–79.3)
	Llama 3.1–8b ‡	85.5 (82.0–88.3)	81.5 (81.0–82.1)
	Llama 3.2–1b	83.4 (79.3–86.6)	81.5 (80.8–82.1)
	DeLLiriuM	84.8 (81.8–87.8)	**82.5 (81.8–83.1)** ^a,b^

Abbreviations: AUROC: Area Under the Receiving Operating Characteristic; EHR: Electronic Health Records; EHR-S: Structured EHR; EHR-T: Text EHR; NN: Neural Network. Performance is shown as the median AUROC across 200-iteration bootstrap with 95% Confidence Intervals in parenthesis. P-values are based on pairwise Wilcoxon rank sum tests.

a p-value <0.05 compared to best structured EHR baseline.

b p-value <0.05 compared to best text EHR baseline.

† Best structured EHR baseline.

‡ Best text EHR baseline.

## Data Availability

The data from MIMIC and eICU is publicly available and can be accessed through PhysioNet at the following links after obtaining approval: MIMIC-IV (https://physionet.org/content/mimiciv/2.2/) and eICU-CRD (https://physionet.org/content/eicu-crd/2.0/). The data from UFH is private and has not been approved for public use.

## Code availability

The code for running all data processing, model training/validation, and analyses, is freely available at DeLLiriuM.
